# What Constitutes the Respiratory Changes?

**Published:** 1867-11

**Authors:** Rufus King Browne

**Affiliations:** Prof. of Physiology and Mic. Anat.: N. Y. College of Dentistry.


### ARTICLE II.

### *What constitutes the Respiratory Changes?*

By Rufus King Browne, M. D., Prof. of Physiology and Mic. Anat.: N. Y. College of Dentistry.

[I do not claim in the ensuing description, to present a demonstration of the tissue changes, which I believe regularly take place during the respiratory movement, nor to place beyond doubt the effect which these changes have in inducing the insemination of oxygen into the vascular flow, but to fix attention upon the until now unnoticed existence of the facts I state: and thus to anticipate a further account of experiments, which tend to show the correctness of my belief, or if fully successful to furnish an experimental demonstration of them, as facts in physiology.]

Of the two sets of physical phenomena, which are cited to account for the passage of the oxygen into the blood, neither are satisfactory; for it cannot fail to occur on a moment's reflection, in the mind of the competent physiologist, that the phenomena to be accounted for, do not take place in obedience to the law under which the two former sets of phenomena, supposed to be of identical nature, *do* take place.

Thus, the phenomena of endosmose, and exosmose, in which two fluids of different characters or constitutions, as menstrua at first separated by a septum, or solid partition, diffuse on both its sides, and come together, after previous separation, and the phenomena of the mutual diffusion of different gases, similarly once separated, are not phenomena of the same character, as those which occur in the passage of oxygen into the blood. This latter phenomenon, is one which does not take place, either in the diffusion of gases, or in the osmose or exmose of fluids. True, two of the *component* substances of the transaction, in either case, are similar *substances*, but in fact, the *process* performed on the one hand by the carbonic acid in the blood, and on the other by the oxygen, is *neither* a mutual transfusion of at first separated fluids, nor a mutual diffusion of two at first parted gases. The process, in the first case is really one in which a proportion of one of the two constituents of the air—its oxygen, *separates* from its other constituent, and penetrates to a thick liquid and soft-solid constituent, on the one hand, while a heavy gas *separates* from the same liquid, and penetrates into the remaining disproportionate constituents of the air, from which the oxygen came. The phenomena of osmose are not involved in this process at all, nor are phenomena at all identical with those of the diffusion of gases involved ; and no event *similar* to such gaseous diffusion takes place until after the process of *separating* of a gas from a fluid, and its mingling with a second gaseous substance occurs,—and this transaction is one which occurs, not as that we have to account for does, between the blood, and the gaseous volume, but within the lung cavities, where the two are brought into actual contact, and hence mingle. Than this latter transaction, although it involves two of the substances, involved in the other, nothing could be really more *unlike* the respiratory process. For this process is one, briefly, in which a gas naturally in combination with a certain quality of blood, leaves its medium of transport, and *separates* from it, while a portion of a certain volume of gas leaves and separates from its natural combinations with another, each of which takes its respective place and combines separately; in the first case, and on the one hand, effecting, not a diffusion or transient uniting of two *separated* gases, but a *separation*

of one gas from a fluid and the separation of a portion of a second gas, and its immediate uniting with a fluid.

The respiratory process, therefore, is not a mingling of gases, coming together through certain means of separation, namely, a septum, or partition, neither is it a process which in itself occurs in passive fulfilment of any "law" of diffusion of gases. The process is *not* one in which a measure of two gases *diffuse,* and thus constitute a mutual *fusion* throughout the bounds of their respective volumes, equally on either side their dividing line or partition of substance; in short, the process of respiration is one for the *separation* of gas from a fluid, and one gas in part from another, and hence it is not a *diffusion* of gases, and can be neither explained nor understood by a perception of that law. Of course in drawing this distinction;—in showing how widely unlike the two sets of phenomena are, we do not dispute the prevalence of the law of diffusion, but on the contrary, unreservedly accredit and admit it, but we show, that the phenomena themselves, are widely different. The gas which separates from the blood, subsequently of course, diffuses with the combined gases of the air in the lung cavities, *in accord* with that law, and would do so if there were septa put between, across the calibre of the lung cavities at the level where they commence to mingle. But this mingling is always consequent upon prior fulfillment of the respiratory process, which consists in the penetration to the blood of the oxygen, and the penetration *from* the blood, of the carbonic acid gas.

In presence of these considerations it seems a demonstrated fact, that the universally accredited explanation of the respiratory process, is a complete misapplication of theory, for it neither involves nor implies any *account of* the very phenomena, in which the process itself consists. It merely takes account of the behaviour of certain gases or substances engaged in a certain process, not in the smallest particular like the process we wish to account for. In fact, the so-called law of "diffusion" of gases, is simply *nominal,* being nothing more nor less, than the *fact* of the mingling or diffusion of certain measures of different gases *even where* a solid septum intervenes between the two. While the respiratory phenomena so far as the same substances are involved, consist in the separation or abstraction of a gas in a natural state of combination in a viscid medium *from* that medium, in spite of the presence of a tube which hermetically holds both, and, secondly, of the passing of a certain amount of oxygen in association with nitrogen, through the whole of the same tube into the blood.

The consideration of these facts has long impelled me to find an explanation of the respiratory process in accord with the facts. Certain general considerations present themselves, of which we may here take note.

All physiology is a *process,* and *consists of a progressive variation* in what would otherwise be a *state,* that is a condition following the association of a process.

It is then a "*process*" we have to understand.

Without exception, all bodily processes, are based upon anatomical conditions, i. e. the character and arrangement of the tissues. The impulsion of the blood, is dependent on the tissues and the change in *movement* of the heart. This motion of the heart is a change in position of every anatomical element composing it. The act of digestion is a movement dependent on the movement of the stomach. This movement is a change in position of all its anatomical elements.

In the respiratory process the lungs are continually passing from one state or condition to another, without cessation,—from one state in which their tissue is of the least, to one, in which it is of the greatest magnitude. This progressive change in the feature of the entire lung, involves a change in the feature of its various anatomical component elements. At their regular minimum size, the lungs do not occupy the whole of the pleural or thoracic cavity: at their maximum size they completely fill it, and extend it by descending upon the abdominal cavity. This regular, constant, and reciprocal progression involves a change of feature and size of even its minutest elements. The entire change in feature and magnitude of the whole lung is only the aggregate of the change of size and figure of each of its elements. Of course this progressive alternation, will most change the figure of the anatomical elements of the lung most easily changed. In the extreme or terminal portion of the lung, there ramifies the most compact capillary system, yet found in the human body, and the readily yielding sides of this part of the tissue would subject them to the greatest part of this change in enlargement and diminution of lung tissue. The movement of the lungs is *un*remitting. From the moment it reaches its greatest dimensions to recur to its least dimension, and again from the latter to the former, there is a progressive movement. The moment of the greatest size, from which the lung recedes, and the moment of least size, from which it *re*enlarges, are not periods of cessation, but *stages* of the movement. And it is during these stages of the movement, that the greatest change in the figure of the vascular channels is made, and at the movement continuing from these stages, either toward decrease or increase of size —there is the *mean* anatomical state of these vessels.

The capillary net-work of the cellular, vesicular, or saccular portion of the lung-structure, is so close as to leave but slight intercapillary spaces, the outer surfaces of their walls being nearly in contact, and between there is no *matrix* or intercapillary tissue. They ramify upon the *outer* or pleuro-pulmonary surface of the bottom and sides of the air-sacs, and between them up to the dividing plane of the sacs. Their position is one in which they receive transversly the full force of any change in the size of the air-sacs.

During the progressive stages of enlargement of the lung simultaneous with the increase in the volume of the increasing air, the air-sacs are enlarged in size and calibre, by increase of calibre both in the direction of their longer axis, and of their shorter. The pressure of the accession of incoming air is borne directly by the capillaries, upon their transverse limits, and thus upon their contents, causing a *remission* or an *impulsion* in their rate of advance. This is the advanced stage of enlargement of the lung-tissue. As the air recedes from the bronchial tube, the tension on the air-cells abates. The return of the air-cells or sacs, to the least diameter and size, pushes the residual air along the bronchial tubes, and the stage of movement is reached in which the lung tissues have their least size. This is the stage of the uniform and uninterrupted flow of the contents of the capillaries to be again, without cessation of movement, changed into the stage of enlargement.

There is thus in the capillary portion of the lung tissue, consecutive remission of rate of flow, in a stage of the onward movement of their contents in which the volume is positively *retarded*, and one in which the retardation is followed, by a *brisker* or more *hurried* flow. Though I have not been able experimentally to determine this point, it is during one of those stages, that the carbonic acid transudes from the capillary walls, and through this epithelium layer and enters their interior, and during the other, that the oxygen is absorbed into the plasma, from which it is imbibed by the red-corpuscles.

But if we continue to follow the process of enlargement of the tissues, we shall convince ourselves with entire certainty, that it cannot take place, without inducing the effects on the capillaries and their contents, we have described. The innumerable branch cavities of the great bronchial tree, are a bundle of air-containing elongated hollows, the extreme or terminal portions of which are the air-sacs or cells. When the entire hollow portions of the lung contain their least volume of air, the tissue of the cell is at its *least* tension ; with increase of volume of air ; the tension caused by the increased volume and pressure of air rises, and is communicated to the capillary structure, which surround them, and ramifies between them. That portion of the capillary web running between is subjected to the pressure on both sides of their walls. On the other side, the pleura or binding coat of the under tissue, offers resistance to the enlargement of the tissue it circumscribes and unites. Even while in the process of enlargement, its resistance to it is being overcome, it exerts more or less pressure upon the tissues it holds. Of these the capillaries would be affected most. We have thus during enlargement a combined pressure upon these blood-holding hollows, from the pleura and from the air which primarily increases the calibre of the air-sacs, and secondarily exerts pressure upon the vessels immediately in contact with them.

That certain anatomical or tissue movement, involving a change in function for the time being in the tissue, is demanded where notable physiological transactions occur, is exemplified in the movements of the heart, the stomach and the intestines. The movement of the heart and the anatomical changes in its tissue, which constitute that movement, alone give the needed impulsion to the blood. And similarly the changes in the tissue of the stomach alone give the needed transudation of the gastic juice. So, also the changes of tissue in the intestines alone furnish the products of that canal, and slowly carry onward the undigested food. To the *movement* of the lungs and the regular tissue changes it involves is to be assigned the transpiration of the carbonic acid absorbed and carried forward by the blood, and the absorption of the proportion of oxygen which enters the blood. A portion of the tissue changes, which take place in the heart, is so nearly similar to those I have described in the lungs that the former suggest the latter. Thus in a shortened or abbreviated diastole, and hence increased frequency of the systole, the tissue of the heart is not permitted to reach its normal limit of enlargement, and hence a diminution of its actual contracted effect.

---

### ARTICLE III.

### *Facts and Philosophy of Dental Progress. No.* 3.

